# Disrupted Signaling through the Fanconi Anemia Pathway Leads to Dysfunctional Hematopoietic Stem Cell Biology: Underlying Mechanisms and Potential Therapeutic Strategies

**DOI:** 10.1155/2012/265790

**Published:** 2012-05-23

**Authors:** Anja Geiselhart, Amelie Lier, Dagmar Walter, Michael D. Milsom

**Affiliations:** ^1^Division of Stem Cells and Cancer, German Cancer Research Center (DKFZ), Experimental Hematology 69120, Heidelberg, Germany; ^2^Experimental Hematology Group, Heidelberg Institute for Stem Cell Technology and Experimental Medicine (HI-STEM) gGmbH, 69120 Heidelberg, Germany

## Abstract

Fanconi anemia (FA) is the most common inherited bone marrow failure syndrome. FA patients suffer to varying degrees from a heterogeneous range of developmental defects and, in addition, have an increased likelihood of developing cancer. Almost all FA patients develop a severe, progressive bone marrow failure syndrome, which impacts upon the production of all hematopoietic lineages and, hence, is thought to be driven by a defect at the level of the hematopoietic stem cell (HSC). This hypothesis would also correlate with the very high incidence of MDS and AML that is observed in FA patients. In this paper, we discuss the evidence that supports the role of dysfunctional HSC biology in driving the etiology of the disease. Furthermore, we consider the different model systems currently available to study the biology of cells defective in the FA signaling pathway and how they are informative in terms of identifying the physiologic mediators of HSC depletion and dissecting their putative mechanism of action. Finally, we ask whether the insights gained using such disease models can be translated into potential novel therapeutic strategies for the treatment of the hematologic disorders in FA patients.

## 1. Introduction

Fanconi anemia (FA) is a rare, autosomal recessive and X-linked hereditary disorder, which is characterized by progressive bone marrow failure (BMF), congenital developmental defects, and an early onset of cancers such as leukemia and some solid tumors [1]. In general, the hematologic manifestations of FA remain the primary cause of morbidity and mortality, with patients suffering from a markedly increased risk of myelodysplastic syndrome (MDS) and acute myeloid leukemia (AML). In addition, FA patients are also predisposed towards various forms of solid tumor such as squamous cell carcinoma of the head and neck, esophagus, and gynecologic area [2, 3].

FA is a genetically heterogeneous disorder caused by inactivating mutations in genes that are thought to function in an epistatic signaling pathway. Loss of function of any of the FA family members results in inefficient repair of DNA damage and deregulation of signaling pathways controlling cell proliferation and apoptosis. To date, 15 genes associated with FA in patients have been identified and cloned: *FANCA, FANCB, FANCC, FANCD1/BRCA2, FANCD2, FANCE, FANCF, FANCG, FANCI, FANCJ/BACH1/BRIP1, FANCL/PHF9/POG, FANCM, FANCN/PALB2, FANCO/Rad51C *[4], and* FANCP/SLX4/BTBD12* (Table 1) [5–7]. The FA proteins appear to function in a common biochemical ubiquitin-phosphorylation network, the FA signaling pathway, that is involved in controlling multiple functions related to DNA repair and the cellular response to stress [8]. Upon DNA damage, FA proteins are recruited to the site of damage and assemble to form the FA core complex. This nuclear multiprotein complex consisting of FANCA, FANCB, FANCC, FANCE, FANCF, FANCG, FANCL, and FANCM functions as an E3 ubiquitin ligase and mediates the activation of the ID complex, which is a heterodimer composed of FANCD2 and FANCI. Once monoubiquitinated, it interacts with classical tumor suppressors downstream of the FA pathway including FANCD1/BRCA2, FANCN/PALB2, FANCJ/BRIP1, and FANCO/Rad51C and thereby contributes to DNA repair via homologous recombination (HR) [9].

Defects in any of the gene products associated with the FA pathway result in similar cellular abnormalities. Firstly, cells isolated from FA patients show elevated levels of chromosomal aberrations and are hypersensitive to DNA interstrand crosslinking agents such as mitomycin C (MMC), cisplatin, diepoxybutane (DEB), and melphalan [17–21]. These DNA alkylating agents covalently link two bases on opposite strands of the DNA and thereby cause replication arrest and DNA double-strand breaks, which ultimately leads to cell death. The increased susceptibility of FA cells to these compounds indicates a defect in the DNA repair machinery that is usually involved in the resolution of these crosslinks. The evaluation of such abnormal structures in response to the clastogenic effect of crosslinking agents provides a reliable cellular marker for the diagnosis of FA and allows the identification of patients presenting with aplastic anemia or leukemia that would not be recognized in the absence of the characteristic physical signs associated with FA. The so-called chromosome breakage test exposes cultured FA cells to alkylating agents such as DEB and MMC, in order to provoke chromosomal abnormalities. While MMC causes radial chromosomes [18], DEB mainly functions as a bifunctional crosslinking agent inducing chromosomal breakage or rearrangements [17]. More recently, the FA pathway has been shown to be involved in the cellular response to DNA damaging agents that do not cause crosslinks. One example is the O^6^-alkylating agent temozolomide, which is commonly used in the treatment of glioblastomas. It has been shown that inactivation of the FA pathway, in particular FANCG and FANCD1/BRCA2, renders cells more susceptible to apoptosis following treatment with temozolomide, suggesting that a functional FA pathway is required for the sensing and/or resolution of the DNA adducts formed by this agent [19, 20].

An additional cellular phenotype, which can be observed in response to the treatment of FA cells with DNA damaging agents like MMC and melphalan, is an exaggerated arrest of cells in the G2/M phase of the cell cycle. Cultured cells exhibit a prolonged G2 phase transit and frequently arrest in G2. This phenotype can be analyzed by flow cytometry and is useful as an additional diagnostic tool for FA [22–24]. Recently, this assay has been further modified to allow the rapid and accurate determination of complementation groups in FA patients using retroviral-mediated gene transfer of FA cDNAs to correct the melphalan-induced G2/M arrest [25, 26].

Finally, cells with a defective FA signaling pathway demonstrate hypersensitivity to the inhibitory action of proinflammatory cytokines such as tumor necrosis factor *α* (TNF-*α*), interferon-*γ* (IFN-*γ*), and macrophage inflammatory protein-1-*α* (MIP-1-*α*) [27, 28]. Both TNF-*α* and IFN-*γ* are produced at abnormally high levels in the serum and bone marrow (BM) of FA patients and are hypothesized to play a role in hematopoietic failure [29, 30]. Although the mechanistic bases for the hypersensitivity of FA cells to IFN-*γ* and MIP-1-*α* remain to be elucidated, it has been shown that murine BM cells defective in the FA signaling pathway produce excessive reactive oxygen species (ROS) in response to treatment with TNF-*α* [31]. These elevated levels of ROS may in turn comprise a source of DNA interstrand crosslinks and therefore provide a direct link between proinflammatory cytokines and the defective DNA damage response seen in FA cells.

Although FA cells are well documented to be hypersensitive to both DNA crosslinking agents and to pro-inflammatory cytokines, it has not been formally demonstrated whether these two phenomena are related to each other. Perhaps more importantly, the exact identity of the key players driving BMF in FA patients remains unclear. In this paper we summarize evidence that the hematopoietic problems associated with FA are rooted in a stem cell defect and aim to highlight research that reveals insight into the mechanisms through which this stem cell depletion is mediated. We also discuss how this mechanistic data can be used as a starting point to identify new targets for therapeutic intervention.

## 2. Hematologic Abnormalities in FA

Hematologic abnormalities, which are found in virtually all FA patients, include cytopenias such as thrombocytopenia (abnormally low platelet counts in the peripheral blood), neutropenia (low neutrophil counts), and progressive pancytopenia (abnormality in two or three blood cell lineages) [3]. At birth, FA patients usually do not show any signs of these defects and have normal blood cell counts, but, as the patient grows older, the hematologic complications start to develop, mainly within the first decade of life. Macrocytosis (enlargement of red blood cells) is usually the first to be detected, followed by thrombocytopenia and aplastic anemia (insufficient production of red blood cells, leukocytes, and platelets in the BM), finally resulting in the characteristic progressive BMF phenotype [3, 32, 33]. Unless treated, BMF represents the primary cause of morbidity in FA patients.

In addition to the observed low levels of mature hematopoietic cells across all lineages, FA patients have also been found to possess severely compromised hematopoietic progenitor compartments. Growth of BM hematopoietic progenitors from FA patients has been shown to be impaired as they are compromised in their ability to produce colony-forming unit granulocyte-macrophage (CFU-GM), burst-forming unit-erythroid (BFU-E), and CFU-granulocyte, erythroid, monocyte, megakaryocyte (CFU-GEMM) colonies in semisolid culture media *in vitro* [34, 35]. While hematopoietic progenitors of normal individuals, or even patients with aplastic anemia, respond to recombinant stem cell factor (SCF) by improved colony output, FA progenitors largely fail to respond to stimulation with SCF [34]. In a separate study, knockdown of FANCC expression in human BM cells using antisense oligonucleotides resulted in significantly reduced clonal growth of erythroid and myeloid progenitor cells *in vitro* [36]. Taken together, these data clearly implicate a defect at the level of FA progenitor cells.

As well as suffering from the progressive depletion of normal BM cells, FA patients are also predisposed towards malignant transformation. Approximately half of all FA patients present with MDS and/or AML before they are 40 years old [33]. Both of these disorders result from the dominance of abnormal hematopoietic clones, likely caused by genetic instability within the hematopoietic stem and/or progenitor compartments.

## 3. The Evidence for Hematopoietic Stem Cell (HSC) Defects in Patients

Since all hematopoietic lineages are compromised in FA patients, it would seem reasonable to assume that a defective FA signaling pathway may negatively impact upon the biology of HSCs, which comprise the top of the hematopoietic system hierarchy. However, while it is relatively straightforward to assess the depletion of mature hematopoietic cells and myeloid progenitors in FA patients, it is more difficult to directly examine HSC function. Nonetheless, there are several lines of evidence in FA patients that suggest that the HSC pool is compromised.

Firstly, at the immunophenotypic level, FA patients demonstrate significantly decreased frequencies of BM CD34+ cells, which are comprised of the HSC and progenitor compartment. As this is also true for patients that do not yet show any evidence of BMF, this finding supports the hypothesis that FA patients have a defect at the level of the stem cell [37]. Importantly, this correlates with the finding that there are reduced stem/progenitor numbers in umbilical cord blood taken from newborn FA patients [38]. Thus, HSC/progenitor depletion appears to precede the decreased production of mature lineage committed hematopoietic cells and may have already begun during early development.

Secondly, the BMF syndrome can be corrected by allogeneic HSC transplantation using minimal conditioning [39, 40]. Thus, in a mixed chimera setting, normal donor HSCs are able to permanently reconstitute the failing hematopoietic system to a corrective level, regardless of the environment of patient FA hematopoietic and niche cells that the donor cells are infused into. As well as eliminating the possibility that the FA HSC niche has a dominant role to play in progressive BMF, this also suggests that FA HSCs are defective relative to their normal counterparts.

 Finally, the phenomenon of reverse mosaicism reveals that the FA defect can be corrected with a single functionally normal HSC. Reverse mosaicism occurs as a consequence of the correction of one of the patient's nonfunctional FA alleles within a somatic cell clone. In FA patients, this correction has been documented to arise from either the genetic recombination of two compound heterozygote mutations in a FA gene, resulting in the production of a single functional allele; or from spontaneous point/frame shift mutations that restore function to the inactivated gene [41–43]. Importantly, the corrected clone must demonstrate a survival advantage over its noncorrected counterparts in order to be able to expand to sufficient numbers to facilitate detection and confer a therapeutic effect. This survival advantage must be present in FA patients, as in several independent instances, a single corrected hematopoietic clone has been shown to support the hematopoietic function and survival of the patient, despite the fact that cells within other somatic tissues did not contain the correcting mutations [41, 42]. Although these spontaneously reverting clones have been documented to persist for a number of years in several patients, it is still possible that the correcting mutation occurred in a long-lived progenitor cell as opposed to an HSC. However, one intriguing study has described the occurrence of reverse mosaicism from a single clone in monozygotic FA twins. Although the twins presented with nonhematologic symptoms of FA and their skin fibroblasts were sensitive to DNA crosslinking agents, their hematopoietic cells appeared to function normally [43]. Upon molecular analysis, it was revealed that the twins both possessed a revertant clone with exactly the same correcting mutation. Presumably, a single somatic hematopoietic cell must have been subject to this correcting point mutation *in utero,* and then its descendants must have been distributed between both twins during gestation. Since this single clone was able to reconstitute the hematopoietic system of both twins for more than two decades, the original mutation must have occurred within an HSC. This observation clearly demonstrates the selective advantage of corrected FA HSCs over noncorrected HSCs and therefore reveals a survival defect at the level of HSCs in FA patients.

## 4. Model Systems to Study FA HSCs

Clinical observations from FA patients provide some evidence that allows us to implicate a defect at the level of HSCs in driving the BMF disease phenotype. Nonetheless, experimental model systems must be employed to directly interrogate the function of HSCs defective in the FA signaling pathway in a reproducible manner. The “gold standard” for assessing the capacity of HSCs to be able to differentiate to form all mature lineages of the hematopoietic system, while also being capable of self-renewal, is to perform BM transplantation and measure long-term multilineage engraftment within the recipient. Ideally, this assay would be performed with a limiting dilution of HSCs and involve at least one serial transplantation into a secondary recipient. While this rigorous assessment clearly cannot be performed in patients, a number of surrogate assays have been developed in order to dissect human HSC biology.

 As briefly discussed above, it is possible to measure the frequency of a range of human hematopoietic progenitor compartments by their ability to form colonies in semisolid media supplemented with various hematopoietic growth factors. However, in the case of FA, while a defective HSC pool may cause a decreased frequency of progenitor cells, this assay does not allow the direct evaluation of whether this is the case, as it only enumerates the downstream progeny of HSCs. One technique that has been developed to measure the frequency of more primitive cells based on their ability to maintain the output of hematopoietic progenitor cells during extended periods of *in vitro* culture is the long-term BM culture assay, also known as Dexter culture [44–46]. This approach has been applied to the study of patient-derived FA HSCs with varying results. While Butturini and Gale were able to demonstrate that FA BM cells were able to generate long-term cultures that were able to initiate secondary long-term cultures with robust output of differentiated myeloid cells, Martinez-Jaramillo et al. found that long-term cultures seeded with FA patient marrow were drastically curtailed in terms of output of myeloid and erythroid progenitor cells [47, 48]. These differences likely reflect either disparities in the culture conditions employed, which FA HSCs may be particularly sensitive to, or to interpatient variation, for example due to differences in the degree of BMF at the time of BM biopsy. Nonetheless, these two studies clearly serve as a proof of principle for the use of this approach in assessing FA HSC function and may be useful in the evaluation of potential novel therapeutic strategies using primary patient material.

 While the long-term BM culture approach can be successfully employed as a surrogate assay for HSC function, it does suffer from some critical drawbacks. Hence, to date, it has proven extremely difficult to engineer an *in vitro* system that encompasses the extrinsic cues that maintain HSCs within their *in vivo* niche. One possible solution to this problem is the murine xenotransplantation system. This approach takes advantage of the fact that HSCs derived from human umbilical cord blood, BM, and mobilized peripheral blood can be successfully engrafted into immune-deficient mice [50–53]. To date, there have been no reports of successful direct transplantation of FA patient HSCs using the immune-deficient mouse models that are currently available. However, Cohen-Haguenauer and colleagues were able to demonstrate long-term engraftment of retroviral gene corrected CD34+ cells from an FA patient in complementation group A, using the nonobese diabetic/severe-combine immunodeficiency (NOD/scid) immune-deficient mouse model as a recipient [54]. Moreover, a second group took advantage of the “humanized” NOD/scid IL-2R*γ* ^−/−^/SGM3 (NSG/SGM3) mouse model, which combines a profoundly immune-compromised background with transgenic expression of the recombinant human growth factors SCF; granulocyte macrophage colony-stimulating factor (GM-CSF); interleukin-3 (IL-3), in order to facilitate robust engraftment of FA patient AML cells [55]. Taken together, these studies suggest that it may be possible to develop a human FA HSC xenotransplant system given an appropriate murine recipient model and an adequate supply of patient CD34+ cells. Unfortunately, given the fact that FA patients tend to already be suffering from marrow hypoplasia upon diagnosis, it is unlikely that this approach will be widely accessible to the research community.

 A potential novel source of FA HSCs for use in the laboratory is through the directed differentiation of patient-specific induced pluripotent stem (iPS) cells. A recent study by Tulpule et al. showed that *in vitro* differentiation of FA-deficient pluripotent cell lines, in this case human embryonic stem (ES) cells knocked down for either FANCA or FANCD2, can be used to show a developmental defect in hematopoietic specification, thereby highlighting pluripotent cell lines as a valuable tool to study FA [56]. However, there are two major barriers to the application of iPS cell technology in the study of FA HSCs. Firstly, there are currently no robust protocols available for the directed differentiation of *bona fide* HSCs from either human ES or human iPS cells [57]. The second barrier is it appears that the absence of a functional FA signaling pathway restricts the attainment of pluripotency when somatic tissues are reprogrammed using the approach devised by Takahashi and colleagues, namely, via the retroviral delivery of exogenous OCT4, SOX2, KLF4, and c-MYC [58, 59]. However, recent work from Müller and colleagues would seem to suggest that somatic cells from FA patients can be successfully reprogrammed to a state of pluripotency, albeit at lower efficiencies than normal counterparts, by incorporating slight modifications to the reprogramming procedure, such as reducing oxygen tension [60].

Recently, an alternative cellular reprogramming approach was established for the generation of transplantable HSC/progenitors from normal adult somatic tissues. It was demonstrated that it is possible to convert human fibroblasts directly to multipotent hematopoietic progenitors via OCT4-dependent cellular programming without transiting through a pluripotent state [61]. Notably, using this approach, the authors could demonstrate multilineage engraftment potential using the murine xenotransplant model. It would be intriguing to establish whether this approach could be used to directly bypass the reprogramming deficiency that FA patient fibroblasts suffer from when they are subject to the iPS cell-derivation methodology.

## 5. Alternate Model Systems for FA

Studying the etiology of human disease ideally involves the use of human model systems. However, given the previously discussed constraints that are associated with studying HSC biology in FA, namely, the lack of an abundant source of patient HSCs to act as a starting material and the absence of a transplantation system to assess HSC function, it has been necessary to develop animal model systems for this disease. Fortunately, the FA signaling pathway has been well conserved throughout evolution; thus, there are several potential model systems available (Table 1).

Five of the 15 human genes can be found in plants, including components of the core complex (FANCM and FANCL), the FANCD2 component of the ID complex, as well as the downstream effectors FANCD1 and FANCJ [62]. Orthologs for FANCM can also be found in *Saccharomyces cerevisiae* (MPH1) and Archaea (Hef), while six of the FA proteins have clear orthologs in *Caenorhabditis elegans*: BRC-2 (FANCD1/BRCA2), FCD-2 (FANCD2), FNCI-1 (FANCI), DOG-1 (FANCJ), FNCM-1 (FANCM), and HIM-18 (FANCP/SLX4/BTBD12) [12, 13, 16]. In the genome of *Drosophila melanogaster*, six orthologs of the FA complementation groups are encoded: Brca2, Fancd2, Fancl, Fancm, Rad51C and Mus312 (FANCP/SLX4/BTBD12) [12–14]. Meanwhile, in vertebrates, the chicken DT40 B cell line has been extensively used to study the effects of loss of function in the FA signaling pathway, including inactivation of FANCG, FANCD2, FANCC, FANCI, and FANCP [63–68]. Notably, the complete FA gene family can only be found in vertebrates; thus, the DT40 cell line gives a good system to study molecular interactions of the whole FA pathway [69]. Although these model systems are suitable for pathway analysis, a hematopoietic transplantation system is required for the study of HSC biology.

## 6. Transplantation Models for HSC Research in FA

There are a number of vertebrate model systems that have been used to interrogate HSC biology. In the zebrafish, transplantation of whole kidney marrow cells into lethally irradiated recipient fish was shown to be radioprotective, specifically rescuing the ablation of the hematopoietic system that is observed in nontransplanted fish [70]. This demonstrated that transplantable zebrafish HSCs were to be found in the adult kidney. The recently identified existence of histocompatibility genes in the zebrafish has allowed the further improvement of this transplant system [71]. Although zebrafish contain the full complement of FA family members found in humans, loss of function models have only been described for a few complementation groups [11]. The knockdown of the zebrafish ortholog of FANCD2 using an antisense morpholino approach leads to similar developmental defects as those observed in some FA patients, including decreased body size, microcephaly, and microphthalmia [72]. This suggests that the FA pathway plays a similar role in zebrafish and humans. While the morpholino approach is particularly useful for the study of a gene product during development in the zebrafish, it is not appropriate for the ongoing evaluation of gene function in the adult organism. To date, zebrafish mutant lines for FANCL and FANCD1 have been described [73]. Although these fish have an interesting defect in sex determination, they have no documented defects in hematopoiesis. Nonetheless, it is possible that a more detailed assessment of the HSC function of these mutant fish using the transplantation assay described above may yield a phenotype.

The mouse is by far the most frequently used model system employed to study mammalian hematology. The transplant system for mice is well established, and the immunophenotypic identification of murine HSCs and their committed progeny has dramatically improved over the last decade such that HSCs can be prospectively purified to a frequency of around 1 in 4 cells using flow cytometry [74, 75]. A number of murine models for FA have been developed. Knockout models exist for components of the FA core complex (FANCA, FANCC, FANCG, FANCF, and FANCM), FANCD2, and the downstream effectors FANCD1 and FANCP [5, 76–78]. As in FA patients, a number of common cellular phenotypes are present across the different mouse models. These include defective regulation of the cell cycle and apoptosis; spontaneous genomic instability including chromosome breakage and radial chromosomes; increased sensitivity towards DNA interstrand crosslinking agents such as DEB, MMC, and cisplatin [79]. Our discussion will focus on the hematologic defects in these mice, but a detailed overview of the nonhematopoietic phenotypes can be found in [80]. 

**Figure 1:**
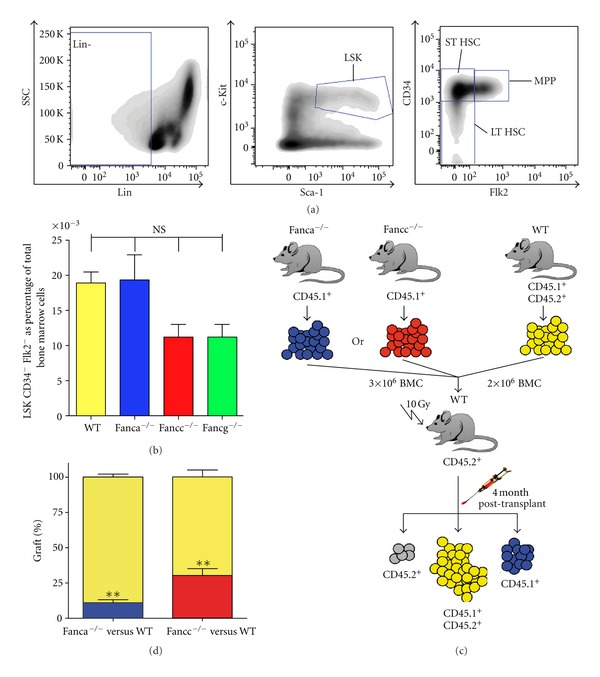
Murine models of FA do not have reduced numbers of immunophenotypically defined HSCs but do have lower frequencies of functionally defined HSCs. (a) Representative FACS plots showing the gating scheme which is employed to enumerate long-term HSCs (LT-HSC) in the BM which are defined by the following combination of immunophenotypic markers: Lineage-, c-Kit+, Sca-1+, Flk2−, and CD34−. For illustrative purposes, the compartments enriched for short-term HSC (ST-HSC) and multipotent progenitors (MPP) are also shown. (b) Based on the FACS methodology shown in (a), the frequency of LT-HSCs does not differ significantly in wild-type (WT) mice compared to FA mice. The mean frequency of immunophenotypically defined LT-HSC found in the BM of WT (*n* = 21), *Fanca* ^−/−^ (*n* = 12), *Fancc* ^−/−^ (*n* = 6), and *Fancg* ^−/−^ (*n* = 6) mice is shown, ± SEM. NS = *P* > 0.05 by comparison using ANOVA. (c) Schematic representation of the competitive repopulation assay employed to assess the relative frequency of HSCs in WT versus either *Fanca* ^−/−^ or *Fancc* ^−/−^ mice. BM from FA (3 × 10^6^ total BM cells) and WT (2 × 10^6^ total BM cells) mice were coinjected into lethally irradiated (10 Gy total body irradiation) recipient mice at a 3 : 2 ratio, respectively. At four months posttransplant, peripheral blood was harvested from recipient mice and the percentage contribution of FA cells to the peripheral blood was determined by FACS analysis, taking advantage of the differential expression of CD45 subtypes on the surface of FA (CD45.1+ and CD45.2−) and WT (CD45.1+ and CD45.2+) leukocytes. If FA BM contained the same number of functionally defined HSCs as WT BM, then the FA HSCs would be predicted to contribute to 60% of the peripheral blood chimerism at 4 months posttransplant. (d) FA HSCs have a severe engraftment defect compared to WT HSCs. The mean relative frequency that FA or WT cells contributed to peripheral blood leukocyte engraftment at 4 months post-transplantation is shown ± SEM. ** = *P* < 0.001 for comparison of WT versus FA chimerism using ANOVA. *n* = 21 for WT versus *Fanca* ^−/−^, and for WT versus *Fancc* ^−/−^. *Fancc* ^−/−^, *Fancg* ^−/−^ and *Fanca* ^−/−^ mice have all been previously described [49].

Of the models for FA core complex loss of function, the phenotypes of FA core complex *Fanca* ^−/−^, *Fancc* ^−/−^, and *Fancg* ^−/−^ mice are almost identical [78]. Their splenocytes and BM progenitors show MMC hypersensitivity. In addition, the BM progenitors are also hypersensitive towards IFN-*γ* and TNF-*αin vitro* and* in vivo *[28, 49, 81–85]. The peripheral blood is normal in *Fancc* ^−/−^ and *Fancg* ^−/−^ mice; however, *Fanca* ^−/−^ mice show mild thrombocytopenia in young (8–10 weeks), but not in older mice [86]. Regarding the BM, *Fancg* ^−/−^ mice demonstrate a defect in the proliferation of mesenchymal stem/progenitor cells and a compromised ability to promote HSC engraftment *in vitro* or *in vivo* [87]. Although *Fanca* ^−/−^ and *Fancc* ^−/−^ mice do not demonstrate reduced numbers of HSCs as determined by flow cytometry analysis, they do have impaired proliferation of progenitors *in vitro* and show a decreased long-term repopulating ability of HSCs in competitive transplantation assays *in vivo *(Figure 1), which may be related to the development of BMF in FA patients [88–90]. Relating to this, *ex vivo* expanded *Fancc* ^−/−^ HSCs demonstrate a dramatically reduced repopulation ability indicating impaired HSC maintenance during stress [91, 92]. Notably, the HSC phenotype of *Fanca* ^−/−^ and *Fancc* ^−/−^ mice can be corrected via retroviral-mediated delivery of the corresponding FA cDNA, formally demonstrating that the defect in the FA signaling pathway is responsible for this phenotype [93–95]. 

Of the two other murine models which are knocked out for components of the core complex, neither the *Fancf* ^−/−^ nor the *Fancm* ^−/−^ mice have been shown to have any hematologic defects to date, although extensive analysis of the HSC compartment using transplantation assays may not have been performed [76, 77].

Two independent murine *Fancd*2^−/−^ models have been developed [96, 97]. *Fancd*2^−/−^ mice have a reduced HSC content, leading to significantly reduced frequencies of late-developing cobblestone area forming cells *in vitro* and defective short- and long-term repopulating ability *in vivo* [96]. Of potential importance is the fact that both *Fancd*2^−/−^ models demonstrate a reduction in the frequency of quiescent HSCs, which may relate to their reduced engraftment capacity. Interestingly, treatment with the antioxidant resveratrol is able to partially correct the hematopoietic defects in *Fancd*2^−/−^ mice [97]. 

The mice that harbor a hypomorphic mutation in the FA downstream effector *Fancd1* (*Fancd*1/*Brca*2^Δ27/Δ27^) have a more severe hematopoietic phenotype than the FA core complex knockout mice [80]. The peripheral blood is normal, but the function of the more immature hematopoietic compartment is significantly compromised, including decreased proliferative capacity of the progenitor compartment and a profoundly reduced competitive repopulation capacity of HSCs [98]. Notably, wild-type or gene-corrected *Fancd*1^Δ27/Δ27^ HSCs transplanted into *Fancd*1^Δ27/Δ27^ mice demonstrated a selection advantage, which would appear to recapitulate the phenomenon of reverse mosaicism that has been observed in some FA patients [98, 99].

 Despite the fact that a number of the FA murine models described above do demonstrate a defect in HSC function during BM transplantation, none of the mice suffer from the progressive BMF that is almost universally prevalent in FA patients. As described below, it is possible to provoke a BMF-like phenotype in some of these murine models by the application of stress situations other than BM transplantation, such as *in vivo *exposure to crosslinking agents or oxidative stress [86, 100]. However, the only single gene deleted FA mouse model that appears to demonstrate aspects of spontaneous BMF is the recently described *Btbd*12^−/−^ (*Fancp* ^−/−^) mouse [5]. *Btbd12* is the mouse ortholog of the evolutionarily conserved SLX4 protein, which is a key regulator of nucleases and critical for DNA damage response. These mice are born at sub-Mendelian ratios; have reduced fertility; are growth retarded and suffer from developmental defects including microphthalmia. In addition, cells from *Btbd*12^−/−^ animals spontaneously accumulate chromosomal damage and are particularly sensitive to DNA crosslinking agents. However, of particular import is the observation that *Btbd*12^−/−^ mice are prone to peripheral blood cytopenias and have decreased myeloid and pre-B-cell progenitor content in the BM. The HSC phenotype of these mice has not been reported to date, but it would be particularly informative to assess how this relates to the other murine FA models and whether there is a progressive, spontaneous loss of the HSC pool as these mice age. The fact that *Btbd*12^−/−^ mice more closely mimic the phenotype of FA patients begs the question as to why this is not the case for other murine models of FA. One possibility is that the FA proteins targeted in the other FA mice do not have an equivalent role in humans and mice. While this is certainly possible, the cellular phenotype relating to their DNA damage response; cell cycle defects; proapoptotic phenotype; hypersensitivity to proinflammatory cytokines appears to be conserved across the species barrier. A second possibility is that most of the FA murine models developed to date are in fact hypomorphic. Again, this would not seem to correlate with the abnormal cellular phenotypes that are obtained in these models. A third possibility is that the mice are not exposed to an environmental stimulus that drives BMF. While this possibility will be discussed at some length below, it would not explain the fact that BMF is observed in the *Btbd*12^−/−^ model, which is presumably exposed to a similar environment as all the other FA mice models. Another alternate explanation is that BTBD12 has additional functions outside the FA pathway. As a putative scaffolding protein for DNA nucleases, it could certainly be involved in other forms of DNA repair. Conversely, it is apparent that the phenotypes of the FA murine models become more severe the further downstream within the pathway the targeted gene is proposed to act. While this may again relate to the other knockout models actually being hypomorphic in terms of FA pathway signaling, it is also possible that the downstream proteins, which are directly involved in DNA repair, can retain some function in the complete absence of upstream signaling. In this context, it would be interesting to examine BTBD12 activity in the other existing murine FA models. 

 While the *Btbd*12^−/−^/*Fancp* ^−/−^ mouse is the only single FA gene loss of function model to recapitulate some aspects of the BMF seen in patients, the compound loss of function of *Fancc* ^−/−^ and *Fancg* ^−/−^ also results in spontaneous hematologic defects including BMF, AML, MDS, and complex random chromosomal abnormalities [101]. This would again seem at odds with the idea that the FA signaling pathway is epistatic and suggests that some of the FA proteins have divergent functions. However, an alternative interpretation would be that the single *Fancc* ^−/−^ and *Fancg* ^−/−^ mutations are in fact hypomorphic for HSC function. Clearly this is a very interesting model system that may be used to gain some insight into the role of abnormal HSC biology in BMF.

 While the murine models of FA seem to be the best system currently available for interrogating the HSC defect in this disease, it is possible that they will never fully recapitulate the hematologic disorders observed in patients. Therefore, it may be beneficial for those in the FA research community to develop a large animal model of FA. While such an undertaking would require a significant financial investment, large animal transplant models such as the existing canine and primate systems have already given us unique insights into human HSC biology and have been invaluable in helping to develop novel therapeutic modalities for other diseases [102].

## 7. Cellular DNA Crosslinking Agents and HSC Depletion in FA

 The observation that none of the currently available models of FA fully phenocopies the progressive BMF observed in patients may relate to the lack of an environmental and/or endogenous factor that drives HSC loss. Since FA cells are invariably hypersensitive to DNA crosslinking agents, resulting in cell cycle arrest and apoptosis, it is not unreasonable to focus upon the identification of potential crosslinking agents in FA patients in the search for a physiologic mediator of HSC depletion (reviewed in [103]). 

 Cellular ROSs are one of the chemical entities that are frequently proposed to act as a source of DNA damage in FA cells. Fuelling this speculation is the observation that primary FA cells from patients and murine models of the disease are more susceptible to karyotypic abnormalities induced by oxygen; have a proapoptotic response following exposure to elevated oxygen and/or ROS; are propagated much more effectively under low oxygen tensions [54, 104–108]. Confirmatory evidence is provided by a study in which compound inactivating mutations of FANCC and the ROS detoxifying enzyme Cu/Zn superoxide dismutase (SOD) in mice led to BM hypocellularity [86]. Relating to this, a recent study by Li and colleagues demonstrated a direct functional interaction between FANCD2 and FOXO3a in human lymphoblast cell lines following challenge with H_2_O_2_ [109]. This interaction was specific for treatment with ROS-inducing agents and was absent in cell lines where the FA pathway was nonfunctional. Using a retroviral overexpression strategy, it was also possible to demonstrate that the FANCD2-FOXO3a interaction was involved in protecting cells from oxidative stress via enhancing the induction of antioxidant genes that are direct targets of FOXO3a. Intriguingly, several groups have also shown that the balance of normal HSC homeostasis, including their genetic integrity, is dependent upon ROS levels, suggesting that the ROS-sensitive phenotype of FA cells may represent an exaggerated form of normal HSC behavior [110–115].

 Reactive aldehydes comprise another potential source of cellular DNA crosslinking agent that may deplete FA HSCs *in vivo*. FA deficient cell lines are hypersensitive to both acetylaldehyde and formaldehyde *in vitro*, while exposure of FA proficient cells to acetylaldehyde *in vitro* results in activation of the FA signaling pathway [116]. Moreover, it has recently been shown that in mice which have compound inactivating mutations of FANCD2 and the acetylaldehyde detoxifying enzyme ALDH2, postnatal exposure to ethanol, the metabolic precursor for acetylaldehyde, results in BMF [117]. These *Fancd*2^−/−^ *Aldh*2^−/−^ mice are also predisposed to spontaneously develop acute leukemia, hence further phenocopying the disease progression in patients.

## 8. Replicative Stress and DNA Damage in HSCs

Since DNA synthesis can be considered a form of DNA damage, replicative stress may also be a candidate for HSC depletion in FA. Indeed, in FA competent cell lines, upon induction of replicative stress, FANCD2, FANCM, and the Blooms complex are localized to discrete fragile sites on sister chromatids during mitosis [118, 119]. These fragile sites comprise common chromosomal break points and are also the location at which stress-induced ultrafine DNA bridges form. In human and murine FA-deficient cells, including FA HSC/progenitors, there is an increased number of ultrafine DNA bridges compared to their wild-type counterparts [120]. This correlates with an increased frequency of cytokinesis failure, as assessed by the number of binucleated HSC/progenitors, and an increased rate of apoptosis. Thus, it is hypothesized that the FA pathway is involved in the resolution of these spontaneously occurring ultrafine bridges and that the absence of a functional FA pathway leads to cytokinesis failure followed by programmed cell death, or to genetic instability. Such a mechanism would be an attractive explanation for the progressive BMF seen in FA patients, as HSCs would potentially be depleted as they were induced into cycle.

## 9. Proinflammatory Cytokines as Potential Mediators of HSC Depletion

In addition to an inability to resolve some forms of DNA damage, FA cells are also hypersensitive to the inhibitory action of certain proinflammatory cytokines. Proinflammatory cytokines are potential physiologic mediators of BMF in FA, since HSCs are routinely exposed to a range of proinflammatory cytokines during either infection or as part of the etiology of diseases with an inflammatory component, such as rheumatoid arthritis.

The investigation into the role of proinflammatory cytokines in the hematologic defects found in FA patients was driven by the observation that such signaling molecules are expressed at elevated levels in some FA patients due to aberrant activation of intracellular stress-response signaling [29, 30, 121–124]. Although this abnormal expression pattern could conceivably be a cause or an effect of progressive BMF, subsequent work established that FA-deficient hematopoietic progenitor cells were subject to an inhibition of proliferation and increased apoptosis following treatment with TNF-*α*, IFN-*γ*, and MIP-1-*α* [27, 29, 125–127]. Although there is no mechanistic data to explain the sensitivity of FA cells to MIP-1-*α*, some signaling pathway analysis has been performed to dissect how the inhibitory effect of IFN-*γ* and TNF-*α* on FA deficient cells is mediated.

In FANCC deficient cells, IFN-*γ* exposure results in decreased phosphorylation of STAT1, 3, and 5 and, in the case of STAT1, is the result of FANCC binding to STAT1 and being required for its docking with the IFN-*γ* receptor [128, 129]. Although JAK/STAT signaling is involved in cell survival, the suppression of STAT1 signaling alone in FANCC-deficient cells may not explain IFN-*γ* inhibitory effects since hematopoietic progenitors from *Stat*1^−/−^ mice are resistant to IFN-*γ* treatment [129]. In addition, double-stranded RNA-dependent protein kinase (PKR) is constitutively activated in FANCC^−/−^ cells and demonstrates an increased binding affinity for double-stranded RNA [127]. While overexpression of PKR leads to an increased apoptotic response of FANCC^−/−^ cells to IFN-*γ*, inhibition of PKR signaling partially rescues the hypersensitivity phenotype, thus establishing a role for PKR downstream of IFN-*γ*. In a separate study, overexpression of a dominant negative form of the PKR activator RAX resulted in increased resistance of *Fancc* ^−/−^ murine embryonic fibroblasts to IFN-*γ* [130]. Although the FANCC protein does not directly bind to PKR, it may promote resistance to IFN-*γ*-mediated apoptosis via its interaction with Hsp70 [127]. Intriguingly, Pang and colleagues were able to generate mutant versions of the FANCC protein that were able to correct the crosslink repair defect of FANCC-deficient cells and rescued FANCD2 monoubiquitination yet were unable to facilitate a normal activation of STAT1 and resulted in continued hypersensitivity of the cells to IFN-*γ* and TNF-*α* [131]. This uncoupling of the characteristic DNA repair defect of FA cells and their hypersensitivity to treatment with proinflammatory cytokines suggests that some members of the FA pathway may hold multifunctional roles outside of the canonical function in the sensing and repair of DNA interstrand crosslinks. Whatever its mechanism of action, it would seem that IFN-*γ* can elicit its effect at the level of FA HSCs as well as in progenitors, since pretreatment of either *Fancc* ^−/−^, *Fanca* ^−/−^, or *Fancg* ^−/−^ mice with IFN-*γ* facilitates the depletion of endogenous HSCs to such an extent that wild-type HSCs can be successfully engrafted into these mice without additional myeloablative conditioning [132, 133].

In the case of TNF-*α*-mediated suppression of FA hematopoietic cells, there is some evidence to implicate a link between DNA damage and TNF-*α* signaling. Treatment of *Fancc* ^−/−^ mice with either LPS or TNF-*α* results in an exaggerated production of intracellular ROS within HSC/progenitors that may be linked to the requirement for FANCD2 monoubiquitination in the FOXO3a-mediated suppression of oxidative stress [31, 109, 134, 135]. Elevated ROS expression in response to TNF-*α* treatment correlates with increased DNA damage and HSC/progenitor senescence, which can be rescued by the addition of the ROS scavenger molecule N-acetylcysteine (NAC) or by inhibition of the TNF-*α* signaling axis. Interestingly, *ex vivo* treatment of *Fancc* ^−/−^ HSC/progenitors with TNF-*α* results in a ROS-dependent increase in genetic instability; development of TNF-*α* resistant clones; an increased predisposition towards AML upon transplantation of treated cells into recipient mice [31]. Relating to this, extended *ex vivo* culture of *Fancc* ^−/−^ cells leads to an exaggerated reduction in HSC content via induction of apoptosis in addition to an increased frequency of cytogenetic abnormalities and risk of malignant transformation which appears to be related to an acquired resistance to TNF-*α* [92]. Inappropriate activation of the apoptosis signal-regulating kinase-1/p38 MAP kinase signaling pathway has been identified as mediating the proapoptotic response of *Fancc* ^−/−^ cells to TNF-*α*, although it is not clear whether this is downstream of the induction of DNA damage [134, 136]. TNF-*α* would also appear to exert its effect upon FA HSCs, since inhibition of TNF-*α* signaling in *Fancc* ^−/−^ HSCs via ectopic expression of the homeobox transcription factor HOXB4 partially rescues their engraftment defect [137]. Of particular note, TNF-*α* would also appear to act as a negative regulator of wild-type HSC function in mice, again suggesting that FA HSCs act as a hypersensitive version of normal HSCs [138]. Taken together, it would appear that TNF-*α* is able to mediate DNA damage, HSC depletion, and hematologic transformation in the setting of FA-deficient HSC/progenitors.

It is important to note that the majority of the experiments linking proinflammatory cytokines to a role in FA HSC depletion have been performed using only the *Fancc* ^−/−^ murine model. However, the previously discussed studies, which demonstrate an inhibitory role of either TNF-*α* or IFN-*γ* in FA patient-derived cells or in murine models of alternate complementation groups, would seem to suggest that this hypersensitivity may be a generic feature of FA cells [29, 133].

## 10. Current Therapeutic Modalities for FA

Hematopoietic stem cell transplantation (HSCT) derived from the BM, mobilized peripheral blood, or umbilical cord blood of a human-leukocyte-antigen-(HLA-) matched donor currently remains the only curative treatment option for the hematologic abnormalities in FA [139]. In fact, FA was the first disease that was successfully treated by transplantation using cord blood from an unaffected HLA-identical sibling as a starting material [140]. Significant barriers to successful transplantation of FA patients include the challenge of creating a satisfactory preparative regimen in light of the patient's acute sensitivity to chemotherapeutics and radiotherapy [39, 141, 142]; the availability of an HLA-matched donor who does not suffer from the disease. Most patients are dependent on alternative donor grafts from an HLA-unmatched donor as only a very small number of patients have an unaffected, matched sibling donor (less than 25%). However, advances in the conditions used in preparative regimens allow the achievement of almost similar transplant outcomes for both cases, with survival rates of 52–88% for mismatched family members or matched unrelated donors and 69–93% for HLA-identical sibling donor HSCTs [39, 143, 144]. While FA patients are waiting for a suitable HSC donor, supportive care can be provided via red blood cell and platelet transfusions; oral administration of androgens such as oxymetholone, methyltestosterone, the androgen analogue danazol; or the direct injection of growth factors such as granulocyte-colony forming factor (G-CSF) [145, 146]. However, androgen application can also lead to adverse sideeffects like masculinization of female patients, acne, hyperactivity and diverse problems associated with the liver such as deranged liver enzymes, hepatic adenomas and the potential risk of hepatic adenocarcinoma [147]. While hematopoietic growth factors such as G-CSF and GM-CSF are capable of enhancing peripheral blood neutrophil counts, and, in some cases platelets [148, 149], they should be avoided in patients with clonal cytogenetic abnormalities because of the risk of inducing leukemia. In any case, they are only effective for the short-term treatment and HSCT is ultimately the definite therapy.

## 11. Gene Therapy of FA HSCs

Since the lack of availability of HLA-matched disease-free donor HSCs is a major limitation in HSCT, one attractive novel therapeutic modality is the genetic correction of autologous patient HSCs via the reintroduction of the defective FA cDNA using a delivery system such as a retroviral vector. Recent major advances have been made in the field of gene therapy, which has allowed the correction of a range of different inherited genetic disorders with a hematologic basis via the retroviral-mediated delivery of correcting cDNAs into patient HSCs [150, 151]. Since the input cells are patient derived, there should be no issues with immunologic rejection of the graft unless the vector system or transgene payload is immunogenic. The phenomenon of reverse mosaicism, that we have previously discussed, would appear to indicate that FA would be an ideal candidate disease for treatment via gene therapy, since correction of an individual HSC can result in sustained reversal of BMF [43]. Indeed, this finding has been recapitulated in murine models of FA [99]. Unfortunately, FA also presents some unique problems, which means that it may be an incredibly difficult disease to treat with gene therapy using existing technologies. These include the extremely low yield of CD34+ cells that can be collected for gene modification relative to those routinely achieved in non-FA patients; the extreme sensitivity of FA cells to *ex vivo* culture [37, 92]. To date, the clinical gene therapy trials for FA have all failed to achieve robust engraftment of corrected patient HSCs, although advances have been made in the ability of clinicians to transduce FA CD34+ cells with retroviral vectors [37, 152, 153]. Fortunately, some of the model systems that have been developed for FA have been able to assist in the formulation of new strategies that may help overcome the barriers to effective gene therapy of FA.

 The clinical gene therapy trial performed by Kelly and colleagues highlighted the low collection yield of CD34+ cells that are typically obtained from FA patients [37]. Initially, HSC collection was attempted by apheresis of peripheral blood following mobilization with G-CSF. Unfortunately, the cohort of FA patients appeared to be refractory to the HSC-mobilizing effects of G-CSF. However, the ability of murine models of FA to recapitulate this mobilization defect allowed the evaluation of two alternate mobilization protocols. While Milsom et al. were able to show that coadministration of G-CSF with the Rac GTPase small molecule inhibitor NSC23766 is able to rescue the mobilization defect of *Fanca* ^−/−^ HSCs, Pulliam and colleagues were able to demonstrate that the addition of the SDF1-*α*/CXCR4 antagonist AMD3100 to a G-CSF mobilization regimen was able to achieve similar results in both *Fanca* ^−/−^ and *Fancc* ^−/−^ mice [154, 155].

 An alternate route to overcome the barrier of limited patient HSCs being available for genetic manipulation is to enhance the ability of the gene-corrected cells to reconstitute the patient. Murine models have been used to demonstrate that the homing of transplanted HSCs to their niche in the BM following intravenous injection into the recipient is not 100% efficient [156]. The postulation that direct injection of HSCs into the intramedullary cavity might enhance engraftment rates was first demonstrated as a proof of concept using the murine xenotransplantation assay and may be of interest for the treatment of FA patients [157].

In order to reduce the loss of FA HSCs during *in vitro* culture, it would be beneficial to develop a transduction protocol that minimized the *ex vivo* manipulation period. To these ends, lentiviral and foamy retroviral vectors hold the distinct advantage over gammaretroviral vectors in that they are able to efficiently transduce nondividing HSCs and therefore do not require *ex vivo* manipulation protocols that involve a lengthy prestimulation step in order to drive the largely noncycling HSC population to proliferate. Again, using murine models of FA, two groups have independently shown that the use of either lentiviral or foamy viruses to deliver a correcting FA cDNA using a drastically shortened transduction protocol, significantly improves the engraftment capacity of gene corrected cells [158, 159].

One further possibility to completely overcome the problems associated with *ex vivo* transduction of FA HSCs is to perform the stem cell gene transfer *in situ*. The efficacy of this novel approach was demonstrated by intrafemoral injection of a lentiviral vector expressing GFP into adult immune-competent mice. FACS analysis four months after injection showed significant transduction efficiency with up to 12% of the cells, observed in myeloid and lymphoid subpopulations, being positive for GFP. After secondary transplant, 8.1–15% GFP-positive CFU were detectable and integration site analysis confirmed multiple transduced clones contributing to hematopoiesis in these animals [160]. Importantly, Habi and colleagues were able to extend these findings to the FA model and show that intrafemoral injection of a lentivirus encoding the *FANCC* gene resulted in correction of *Fancc* ^−/−^ HSCs *in vivo *[161]. Altogether, this data indicates that *in situ* transduction of adult stem cells using lentiviral vectors is a promising improvement to *ex vivo* gene therapy. Indeed, Frecha et al. have recently improved on this approach, using normal donor and FA stem and progenitor cells to demonstrate that specially modified lentiviral vectors can selectively target human CD34+ cells *in vitro* and *in vivo*. Using a novel vector system, which expresses an envelope pseudotyped with a fusion of SCF and a mutant cat endogenous retroviral glycoprotein, they were able to selectively transduce target human CD34+ cells from nontarget cells in unfractionated total cord blood and BM from FA patients. In addition, the glycoprotein prevents degradation by the human complement system and therefore makes the vector suitable for *in vivo* use [162]. Ultimately, this vector system may provide improved alternatives for gene therapy of FA *in vivo*, as it allowed the selective transduction of human CD34+ cells *in vivo* using the murine xenotransplantation model.

One potential adverse side effect of the use of integrating vector systems to deliver correcting genes into HSCs is the phenomenon of insertional mutagenesis. Insertional mutagenesis arises as a result of the integrated vector causing a change in expression of cellular genes that are proximal to the integration site (reviewed by Muller et al.) [163]. As documented in several clinical gene therapy trials using retroviral vectors, this can sometimes result in deregulated expression of protooncogenes, which can in turn lead to malignant transformation [163]. In the setting of FA, insertional mutagenesis might be particularly important as promalignant mutations caused by the vector integration may synergize with mutations that spontaneously arise as a result of the genetic instability that is inherent to FA cells. However, recent advances in the development of new vector systems that may have less mutagenic potential could prove a way to overcome this problem. These systems include self-inactivating retroviral vectors with weaker promoter enhancer elements [163]; recombinant transposons such as the sleeping beauty retrotransposon [164]; vectors which facilitate the codelivery of zinc finger nucleases in order to facilitate the directed repair of the endogenous FA allele via HR [165, 166]. The latter approach may prove particularly challenging in the setting of FA, where the cells are deficient for some aspects of HR-mediated DNA repair.

## 12. Alternative Novel Therapeutic Modalities Targeting FA HSCs

**Figure 2:**
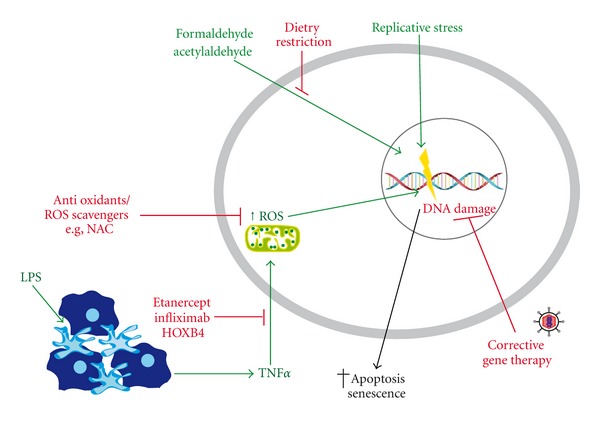
Potential novel therapeutic approaches for the prevention of FA HSC depletion. The potential physiologic mediators of FA HSC depletion, including replicative stress, TNF-*α*, ROS, lipopolysaccharide (LPS), and reactive aldehydes, are depicted as green arrows. Exposure of FA HSCs to these agents/conditions would be predicted to result in DNA damage and ultimately loss of the cell via apoptosis or senescence. Novel therapeutic modalities that directly target these HSC-depleting stimuli are shown in red. Controlling dietary consumption of certain food types (e.g., alcohol) may reduce the production of high levels of reactive aldehyde species in FA patients. Intracellular ROS could be decreased via treatment with antioxidants or ROS scavengers. Proinflammatory cytokine signaling could be targeted by interfering with ligand-receptor binding (e.g., using etanercept or infliximab to block interaction of TNF-*α* with its receptors) or by inhibiting downstream signaling cascades, as demonstrated for HOXB4 overexpression in the context of TNF-*α* signaling. Finally, gene therapy approaches offer the possibility of restoring expression of a functional FA gene into patient HSCs.

In addition to gene therapy, data obtained from experimental models of FA have been used to devise other alternative novel therapeutic modalities for FA (summarized in Figure 2). Metabolism is predicted to generate reactive by-products, such as ROS and aldehydes, which are believed to be one potential physiological source of DNA damage that precipitates the FA phenotype. While healthy cells protect against these threats through the combined action of enzymatic detoxification and DNA repair, FA cells clearly show a defect in this respect.

As previously discussed, there is strong evidence that FA cells are intolerant of oxidative stress as oxygen-induced chromosomal aberrations were observed in cultured FA cells [104] and hematopoietic cells from FA knockout mice exhibit extreme oxidant sensitivity [107]. Therefore, antioxidants promise to improve cell viability by conferring resistance to apoptosis. FANCC-deficient cells pretreated with either selenomethionine or NAC are resistant to H_2_O_2_ treatment and demonstrate enhanced survival [107]. The antioxidant drug resveratrol has been shown to partially correct the hematopoietic defects of FANCD2-deficient mice such as reduced spleen colony-forming capacity and abnormal cell cycle status. Colony forming unit-spleen (CFU-S) assays with whole BM revealed a significantly improved frequency of primitive spleen colony forming cells in resveratrol-treated *Fancd*2^−/−^ mice. HSC/progenitors cells derived from *Fancd*2^−/−^ mice initially showed a significantly lower frequency of cells in G0 when compared to wild-type counterparts; however, resveratrol treatment was capable of increasing the total amount of cells in G0 by 27%. Most importantly, a trial of treating FA patients with a two-week infusion of recombinant human SOD was shown to be effective in decreasing ROS levels. Half of the patients enrolled in this study (two out of four) were less sensitive to DNA crosslinking agents and one had improved BM progenitor numbers [167].

 Recently, it was also found that the FA pathway genes seem to be required in conferring resistance to reactive aldehydes, such as acetaldehyde and formaldehyde [117, 168]. They are thought to be a potential source of DNA damage as they can directly modify DNA bases *in vitro*, which may then lead to crosslinks with DNA or proteins. These findings raise new therapeutic options for the treatment of FA patients. For instance, it may be possible to therapeutically enhance acetaldehyde/formaldehyde catabolic activity using small molecule agonists of the ALDH2 and alcohol dehydrogenase 5, respectively, to provide an intrinsic mechanism for enzymatic detoxification. Based on this data, it would also seem prudent to recommend that FA patients severely restrict their intake of alcohol in order to prevent the accumulation of acetylaldehydes.

A number of FA patients display enhanced serum levels of TNF-*α* and IFN-*γ*, which were previously shown to cause apoptosis of hematopoietic progenitor cells and may therefore be one of the major driving factors in the pathogenesis of BMF [27, 28, 30, 83, 122]. Pharmacologic agents that inhibit the TNF-*α*-signaling pathway, such as etanercept and infliximab, have been suggested as a possible therapeutic intervention directed against the pathogenesis of proinflammatory cytokines and have already proven to be effective in clinical use for the treatment of inflammatory diseases, such as rheumatoid arthritis and Crohn's disease [169]. They hold significant promise for prolonging the hematologic output in FA patients as preclinical models have already demonstrated that inhibition of TNF-*α* using etanercept was effective in increasing the size and number of CFU-E and BFU-E in BM cultures of FA patients [29]. In addition, neutralization of TNF-*α* was shown to be effective in preventing excessive ROS production in FANCC-deficient mice and thereby significantly reduced the DNA damage phenotype as well as senescence [135].

## 13. Summary

 FA is a fatal inherited disorder, which almost universally results in severe defects of the hematopoietic system, likely as a direct consequence of defective HSC biology. Advances in our ability to model the HSC defect in FA patients have not only enhanced our understanding of the underlying etiology of this disease but have also highlighted novel targets for therapeutic intervention. One challenge for the immediate future is to determine whether the defects that have so far been identified in FA HSCs can be extrapolated to explain the abnormal biology of other tissues that are commonly impacted upon by a defect in the FA signaling pathway. This is of particular importance since there are very limited treatment options for the serious nonhematologic complications observed in FA patients such as the increased predisposition towards solid tumors.

Although FA is a relatively rare disease, it is predicted that FA HSCs can, in some instances, act as an extremely sensitive experimental model system to interrogate the biology of normal HSCs. Therefore, understanding the behavior of FA HSCs not only benefits the community of FA patients, but may potentially impact upon our knowledge of more common disorders such as leukemias, myeloproliferative disorders, and other BMF syndromes such as acquired aplastic anemia.

## Figures and Tables

**Table 1:** Members of the FA signaling pathway found in different species.

*Homo sapiens*	*Mus musculus*	*Danio rerio*	*Drosophila melanogaster*	*Caenorhabditis elegans*	*Archaea*	*Saccharomyces cerevisiae*
*FANCA*	*Fanca*	*fanca*				
*FANCB*	*Fancb*	*fancb*				
*FANCC*	*Fancc*	*fancc*				
*FANCD1/BRCA2*	*Fancd1*	*fancd1*	*brca2*	*brc-2*		
*FANCD2*	*Fancd2*	*fancd2*	*fancd2*	*fcd-2*		
*FANCE*	*Fance*	*fance*				
*FANCF*	*Fancf*	*fancf*				
*FANCG*	*Fancg*	*fancg*				
*FANCI*	*Fanci*	*fanci*		*fnci-1*		
*FANCJ/BACH1/BRIP1*	*Fancj*	*fancj*		*dog-1*		
*FANCL/PHF9/POG*	*Fancl*	*fancl*	*fancl*			
*FANCM*	*Fancm*	*fancm*	*fancm*	*fncm-1*	*hef*	*MPH1*
*FANCN/PALB2*	*Palb2*	*palb2*				
*FANCO/Rad51C*	*Rad51*		*rad51C*			
*FANCP/SLX4/BTBD12*	*Slx4*		*mus312*	*him-18*		

References	[10]	[11]	[12–14]	[12, 13, 15]	[16]	[16]
